# H3-T6SS of *Pseudomonas aeruginosa* PA14 contributes to environmental adaptation via secretion of a biofilm-promoting effector

**DOI:** 10.1007/s44154-022-00078-7

**Published:** 2022-12-28

**Authors:** Yantao Yang, Damin Pan, Yanan Tang, Jiali Li, Kaixiang Zhu, Zonglan Yu, Lingfang Zhu, Yao Wang, Peng Chen, Changfu Li

**Affiliations:** State Key Laboratory of Crop Stress Biology for Arid Areas, Shaanxi Key Laboratory of Agricultural and Environmental Microbiology, College of Life Sciences, Northwest A&F University, Yangling, 712100 Shaanxi China

**Keywords:** *P. aeruginosa* PA14, Regulation, H3-T6SS, TepB, Stress resistance, Virulence

## Abstract

Microbial species often occur in complex communities and exhibit intricate synergistic and antagonistic interactions. To avoid predation and compete for favorable niches, bacteria have evolved specialized protein secretion systems. The type VI secretion system (T6SS) is a versatile secretion system widely distributed among Gram-negative bacteria that translocates effectors into target cells or the extracellular milieu via various physiological processes. *Pseudomonas aeruginosa* is an opportunistic pathogen responsible for many diseases, and it has three independent T6SSs (H1-, H2-, and H3-T6SS). In this study, we found that the H3-T6SS of highly virulent *P. aeruginosa* PA14 is negatively regulated by OxyR and OmpR, which are global regulatory proteins of bacterial oxidative and acid stress. In addition, we identified a H3-T6SS effector PA14_33970, which is located upstream of VgrG3. PA14_33970 interacted directly with VgrG3 and translocated into host cells. Moreover, we found that H3-T6SS and PA14_33970 play crucial roles in oxidative, acid, and osmotic stress resistance, as well as in motility and biofilm formation. PA14_33970 was identified as a new T6SS effector promoting biofilm formation and thus named TepB. Furthermore, we found that TepB contributes to the virulence of *P. aeruginosa* PA14 toward *Caenorhabditis elegans*. Overall, our study indicates that H3-T6SS and its biofilm-promoting effector TepB are regulated by OxyR and OmpR, both of which are important for adaptation of *P. aeruginosa* PA14 to multiple stressors, providing insights into the regulatory mechanisms and roles of T6SSs in *P. aeruginosa*.

**Supplementary Information:**

The online version contains supplementary material available at 10.1007/s44154-022-00078-7.

## Introduction

Bacteria have evolved specialized protein secretion systems to deliver proteins into the extracellular space or to neighboring cells, and these systems play key roles in interactions with the environment, competitor bacteria, and host organisms (Cianfanelli et al. 2016). The type VI secretion system (T6SS) is a widely distributed type of proteinaceous machinery that delivers effector molecules directly into the inside of target cells via a one-step process (Zoued et al. 2014). T6SS is structurally homologous to contractile phage tails (Filloux 2009), with a complex structure consisting of a VipA/B outer sheath comprising a valine glycine repeat G (VgrG) trimer, PAAR domain-containing protein, Hcp inner tube, and transmembrane-baseplate complex formed of 13 essential core proteins along with additional accessory proteins (Leiman et al. 2009; Zoued et al. 2014). In the current model, T6SS features dynamic firing cycles including assembly, contraction, and disassembly of a sheath-like structure, followed by expulsion of a cell-puncturing device loaded with multiple effectors (Cianfanelli et al. 2016). ClpV and IcmF, two conserved T6SS components with ATPase activity, are crucial to the reassembly of T6SS structures and the secretion of Hcp, VgrG and substrates (Bonemann et al. 2009; Records 2011). T6SS effectors are transported by interacting with a core component or designated cargo effectors, or by fusing to structural components, known as specialised effectors. Furthermore, VgrG, Hcp, and PAAR play important roles in effector delivery (Durand et al. 2014).

As a tool for protein secretion, T6SS is involved in nutrition uptake, toxin delivery, cell-to-cell communication, interspecies competition, and virulence. T6SS and its effectors play various physiological roles improving the adaptability of bacteria to adverse environmental conditions (Lin et al. 2021; Yu et al. 2021). However, T6SS is an energetically expensive machine that is tightly regulated according to environmental conditions. T6SS is controlled by multiple transcriptional regulators in response to a wide variety of signals including salinity, iron concentration, temperature, and other stressors (Yang et al. 2021). For example, T6SS4 is regulated by OxyR under oxidative stress, triggering secretion of the effectors YezP and TssS, in *Yersinia pseudotuberculosis* (Wang et al. 2015; Zhu et al. 2021). In *Burkholderia thailandensis*, the regulators OxyR and Zur induce T6SS4 to secrete the effectors TseM and TseZ in response to environmental stresses (Si et al. 2017a, b). In *Vibrio anguillarum*, RpoS-regulated T6SS is involved in resistance to hydrogen peroxide (H_2_O_2_) and low-pH stress (Weber et al. 2009).

*Pseudomonas aeruginosa* is a common opportunistic Gram-negative pathogen that is widely distributed in the environment. *P. aeruginosa* has been the focus of intense research due to its prominent roles in several diseases, including septicemia and pneumonia (Chen et al. 2015). The *P. aeruginosa* genome encodes various virulence factors including secretion systems that contribute to its pathogenicity toward several hosts (Bleves et al. 2010). *P. aeruginosa* has three distinct and conserved T6SSs (H1-, H2-, and H3-T6SS), which play crucial roles in competition and pathogenicity by secreting multiple effectors (Mougous et al. 2006; Russell et al. 2014; Sana et al. 2016). The expression and functions of *P. aeruginosa* T6SSs are fine-tuned by regulators of various pathways in response to the environment. For example, in *P. aeruginosa* PAO1, H1-T6SS is upregulated by LadS and downregulated by RetS (Mougous et al. 2006); H2-T6SS is negatively regulated by RpoN, Fur, and CueR during bacterial competition and virulence (Sana et al. 2012, 2013; Han et al. 2019); and H3-T6SS is negatively regulated by Fur in response to the extracellular iron concentration (Lin et al. 2017). In *P. aeruginosa* PAK, H1-T6SS is negatively regulated by RsmA and RetS, impacting bacterial biofilm formation and virulence (Brencic and Lory 2009; Moscoso et al. 2011). All three T6SS types are regulated by RsmA and AmrZ in the highly virulent *P. aeruginosa* PA14 (Allsopp et al. 2017). Given the functional diversity of T6SSs in *P. aeruginosa*, their regulatory mechanisms and effectors must be identified, especially in the relatively less studied but highly virulent *P. aeruginosa* strain PA14.

In this study, we explored the regulation of H3-T6SS in *P. aeruginosa* PA14 and found that H3-T6SS is negatively regulated by OxyR and OmpR. Furthermore, we investigated the functions of H3-T6SS and its effector PA14_33970 (hereafter referred to as TepB) in *P. aeruginosa* PA14. Our results suggest that H3-T6SS and TepB play crucial roles in resistance to oxidative, acid and osmotic stresses, as well as motility, biofilm formation, and virulence in *P. aeruginosa* PA14.

## Results

### OxyR and OmpR negatively regulate H3-T6SS expression in *P. aeruginosa* PA14

**Fig. 1:**
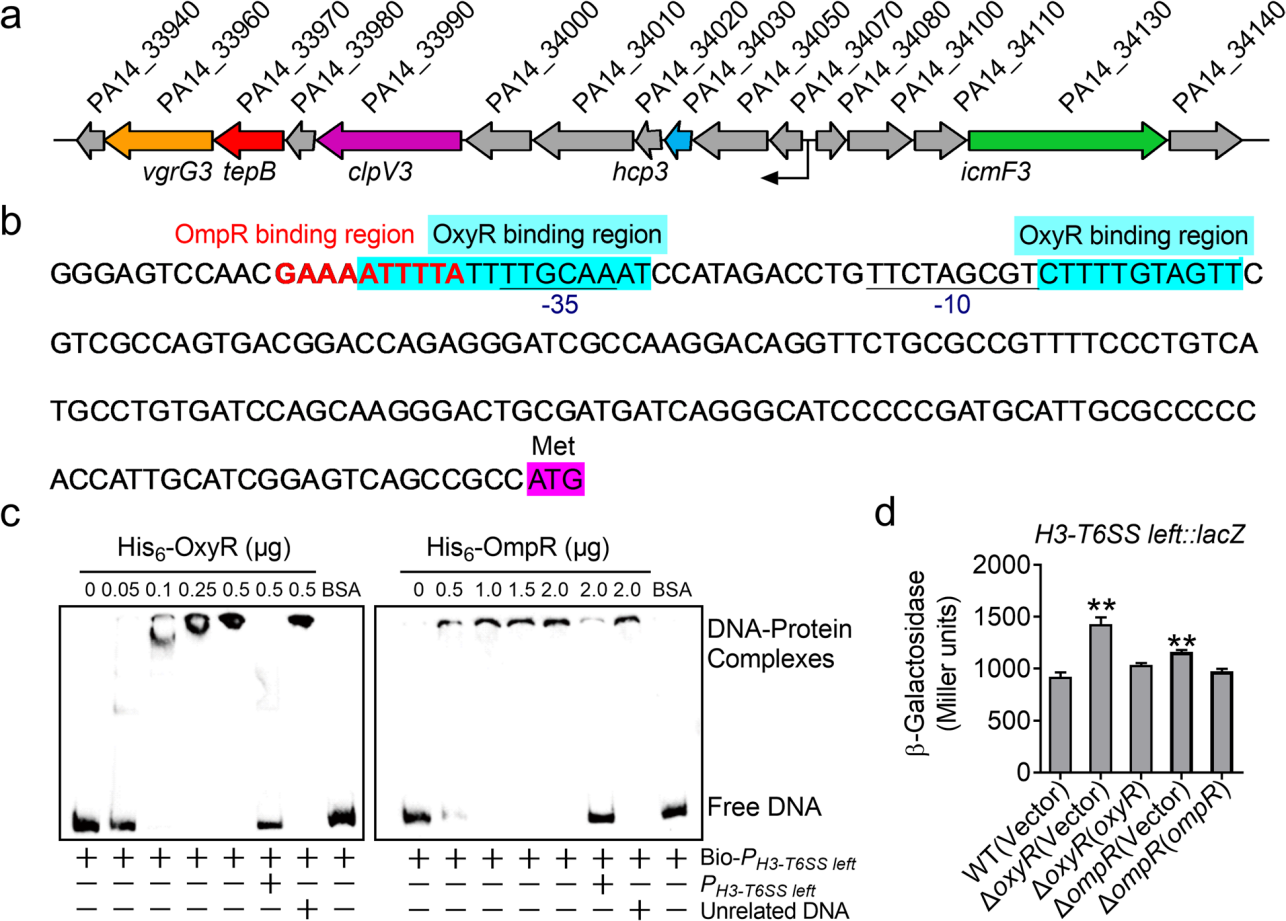
H3-T6SS is negatively regulated by OxyR and OmpR in *P. aeruginosa* PA14. **a** Gene organization of *H3-T6SS* gene cluster in *P. aeruginosa* PA14. **b** The sequence of the *H3-T6SS-left* promoter. The cyan part is the OxyR binding site predicted by software; the bold red letters is the predicted OmpR binding site; the ATG start codon of the first ORF of the *H3-T6SS* operon is marked in purple, and the − 35 and − 10 elements of the *H3-T6SS* promoter are underlined. **c** EMSA experiments verify the binding of His_6_-OxyR and OmpR to the *H3-T6SS* promoter. *P*_*H3-T6SS left*_ is the unlabeled H3-T6SS promoter probe, Bio-*P*_*H3-T6SS left*_ is labeled by biotin, and Unrelated DNA is the unrelated DNA fragment in same length. **d** β-galactosidase analyses of H3-T6SS promoter activities by using the transcriptional *P*_*H3-T6SS left*_*::lacZ* chromosomal fusion reporter expressed in the *P. aeruginosa* PA14 strains grown to stationary phase in TSB medium. Data represent the mean ± SEM of three biological replicates, each of which was performed with three technical replicates. **P < 0.01

To investigate the function of H3-T6SS (genes *PA14_33940* to *PA14_34140*) in *P. aeruginosa* PA14, we first analyzed the H3-T6SS gene cluster. We found that H3-T6SS genes are orientated in different directions (Fig. 1a), and that most genes are distributed in the left gene cluster. Then, we analyzed the left H3-T6SS promoter using Virtual Footprint software and identified two OxyR-binding sites (ATTTTATTTTGCAAAT and CTTTTGTAGTT) and an OmpR binding site (GAAAATTTTA) upstream of the gene *PA14_34070* (Fig. 1b). We then examined the interactions of the left H3-T6SS promoter with OxyR and OmpR using electrophoretic mobility shift assay (EMSA). We generated a probe containing the left H3-T6SS promoter sequence (*P*_*H3-T6SS left*_), which was amplified from base − 207 to − 1 relative to the ATG start codon of the first open reading frame of the left H3-T6SS operon. This probe was incubated with His_6_-OxyR or His_6_-OmpR, leading to the formation of DNA − protein complexes (Fig. 1c). These DNA − protein complexes were completely disrupted by the addition of excess unlabeled probe, but not an unrelated control probe. This pattern indicates that the H3-T6SS promoter interacts with OxyR and OmpR, suggesting that H3-T6SS expression is regulated directly by OxyR and OmpR.

To further investigate the roles of OxyR and OmpR in regulating the H3-T6SS operon, the single-copy fusion reporter plasmid *P*_*T6SS3 left*_*::lacZ* was introduced into the genomes of the wild-type (WT), Δ*oxyR* mutant, Δ*ompR* mutant, and complemented Δ*oxyR*(*oxyR*) and Δ*ompR*(*ompR*) strains. Quantitative analysis of the LacZ activity of the resulting strains revealed that *oxyR* and *ompR* deletion significantly increased in the activity of the H3-T6SS promoter, which was fully restored to the WT level by complementation with corresponding plasmids expressing *oxyR* (pME6032-*oxyR*) or *ompR* (pME6032-*ompR*) (Fig. 1d). This result confirms that OxyR and OmpR negatively regulate H3-T6SS expression. As OxyR and OmpR regulate gene expression in response to stresses, the same experiment was performed under 1 mM H_2_O_2_ oxidative stress, and a similar result was obtained (Fig. S1). Taken together, our findings suggest that both OxyR and OmpR negatively regulate H3-T6SS expression by binding to its promoter in *P. aeruginosa* PA14.

### TepB is a substrate of H3-T6SS in *P. aeruginosa* PA14

**Fig. 2:**
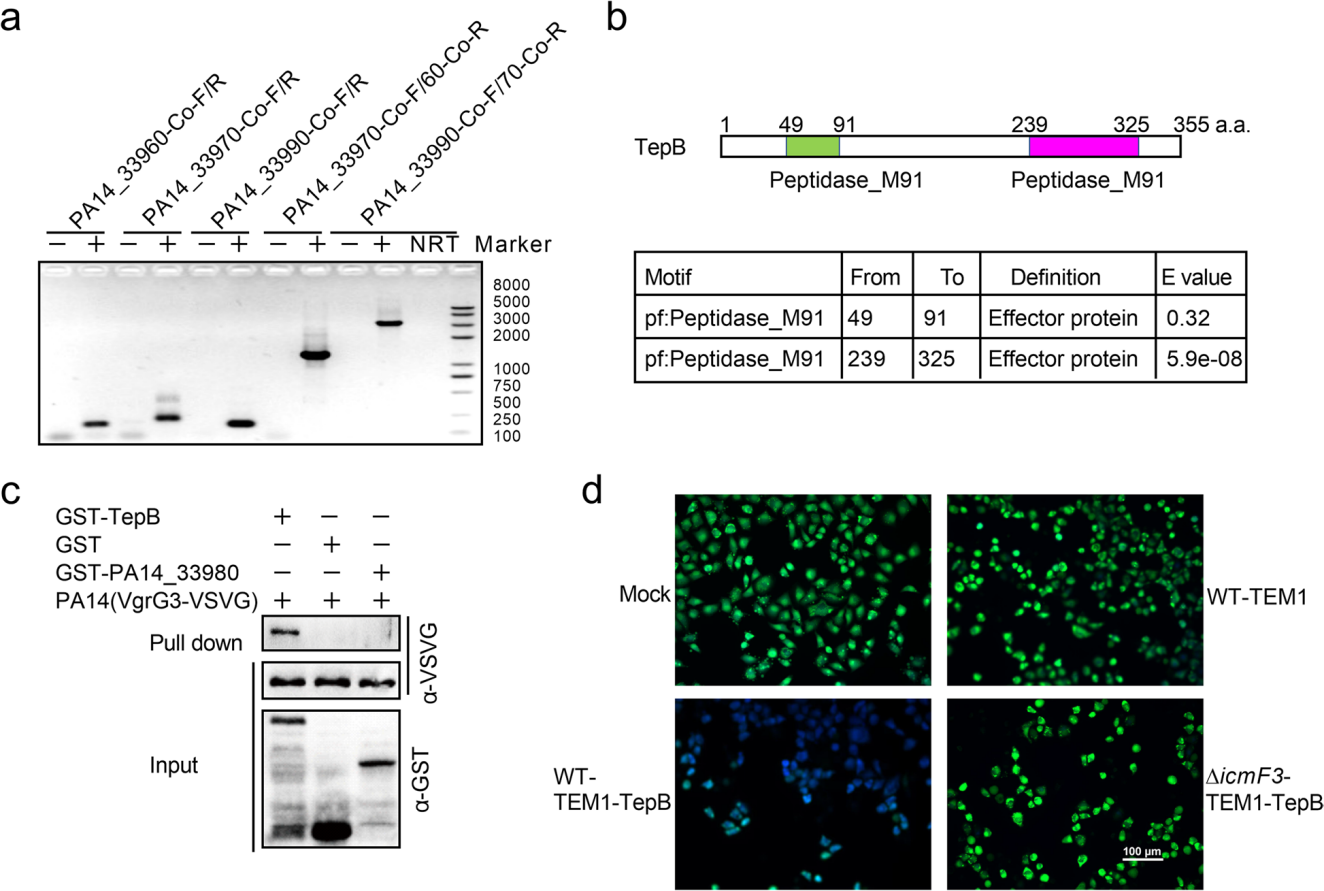
TepB (PA14_33970) is a substrate of H3-T6SS. **a** Cotranscription analysis of *PA14_33970–90* in *P. aeruginosa* PA14 by RT-PCR. -: PCR product with dd H_2_O as template. +: PCR product with *P. aeruginosa* PA14 cDNA as template. NRT: PCR product with no reverse transcriptase sample of *P. aeruginosa* PA14 RNA. **b** Protein motif prediction of TepB. **c** GST pull-down assay to detect the interaction between TepB and VgrG3. VgrG3-VSVG was incubated with GST, GST-TepB or GST-PA14_33980, and the protein complexes captured with glutathione beads were detected by Western blotting. **d** Translocation of TepB proteins into HeLa cells. HeLa cells were mock-infected or infected with indicated PA14 strains expressing TEM1-TepB at an MOI of 100 for 3 h and loaded with CCF2-AM. Visualization of the translocation of TepB using fluorescence microscopy. Scale bar, 100 μm

T6SS is critical to several bacterial processes that involve the secretion of effectors. Genes encoding T6SS substrates of are often located proximally to structural genes such as VgrG and Hcp (Durand et al. 2014; Bondage et al. 2016). Therefore, we examined the putative T6SS effector TepB (PA14_33970), which is located upstream of VgrG3 (PA14_33990) in the H3-T6SS gene cluster of *P. aeruginosa* PA14 (Fig. 1a). We performed reverse-transcription polymerase chain reaction to examine the expression profiles of TepB and the left H3-T6SS gene cluster. Two primer pairs (*PA14_33990*-Co-F and *PA14_33970*-Co-R; *PA14_33970*-Co-F and *PA14_33960*-Co-R) were designed to produce overlapping fragments, designated *PA14_33990-PA14_33970* and *PA14_33970-PA14_33960*. The DNA fragment located between the two focal genes was amplified in reactions containing cDNA, but not in those containing double-distilled water (negative control) (Fig. 2a). This finding indicates that the genes *PA14_33990*, *tepB* (*PA14_33970*)*,* and *PA14_33960* are co-transcribed in the same operon.

TepB is predicted to be an effector protein containing two peptidase M91 motifs (Fig. 2b). Notably, several effector-encoding genes are located in close proximity to the *vgrG3*, *hcp3*, or *paar* gene, and the associated effectors are secreted during interactions with the corresponding protein (VgrG3, Hcp3, or PAAR). Therefore, we performed a glutathione-S-transferase (GST) pull-down assay to examine the interaction of TepB with VgrG3, a H3-T6SS core component that transports secreted effector proteins via direct binding. We found that VgrG3-VSVG was retained by GST- TepB (Fig. 2c). In contrast, no interaction was detected between VgrG3-VSVG and GST or GST-PA14_33980 (Fig. 2c). As reported previously (Jiang et al. 2014; Zhu et al. 2021), translocation of TepB was detected using a TEM1-TepB fusion protein in HeLa cells treated with fusion proteins from WT *P. aeruginosa* PA14, a T6SS-deficient strain or mock treatment. TEM1-TepB was observed in cells infected with the fusion protein-expressing strain but not the T6SS-deficient strain (Fig. 2d), indicating that TepB was secreted into HeLa cells via T6SS. Our results suggest that TepB is a substrate of H3-T6SS in *P. aeruginosa* PA14.

### H3-T6SS and TepB are required for resistance to oxidative, acid and osmotic stresses in *P. aeruginosa* PA14

In addition to its roles in bacterial competition, host infection, and virulence (Xu et al. 2014; Ho et al. 2017; Song et al. 2021), T6SS has important functions in resistance to various environmental stresses including acid, heat, antibiotic, and oxidative stresses (Wang et al. 2015; Yu et al. 2021). This resistance is achieved via the secretion of effectors, which is typically regulated by transcription factors (Yang et al. 2018). For example, T6SS1 in *Cupriavidus necator*, which is regulated by the transcription factor Fur, secretes the lipopolysaccharide-binding effector TeoL to construct outer membrane vesicles in response to oxidative stress (Li et al. 2021). The OxyR-regulated T6SS4 secretes the Zn^2+^-binding effector YezP, which plays an important role in protection against oxidative stress in *Y. pseudotuberculosis* (Zhang et al. 2013; Wang et al. 2015).

**Fig. 3:**
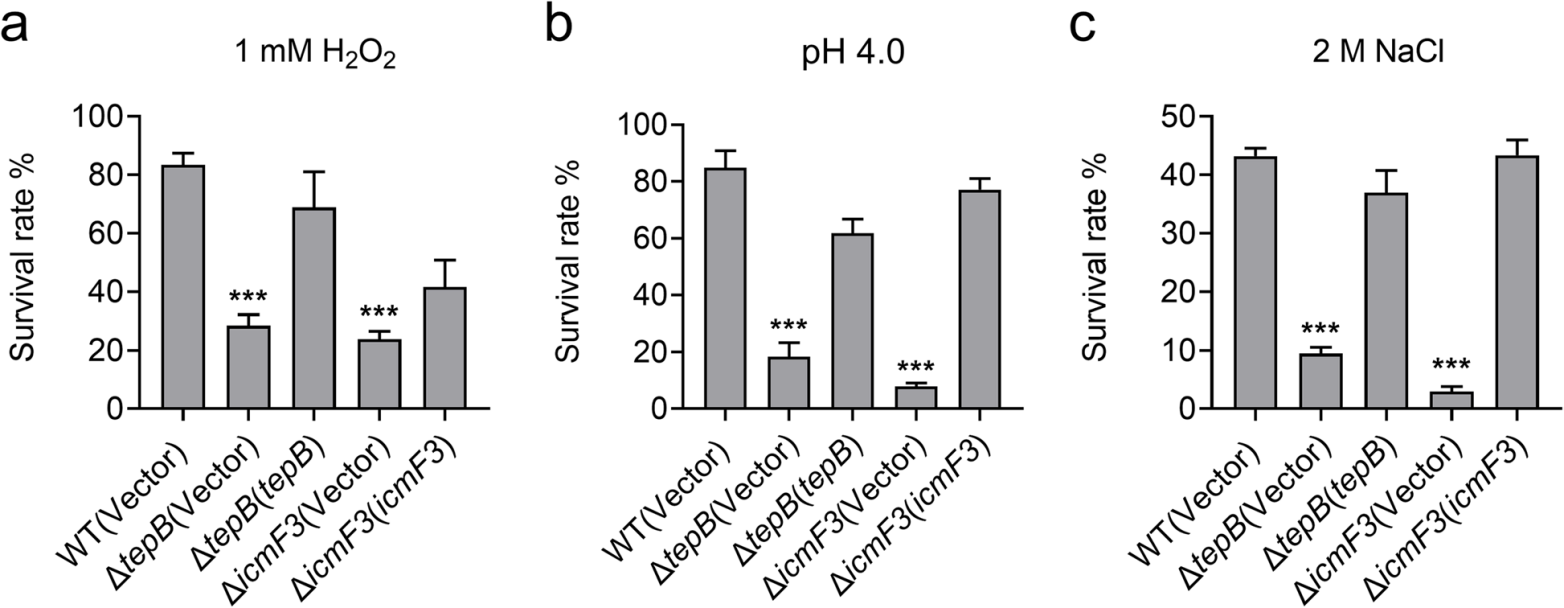
H3-T6SS and TepB are involved in oxidative, acid and osmotic stress resistance. **a**, **b** and **c** The viability of mid-exponential phase *P. aeruginosa* PA14 strains was determined after challenge with 1 mM H_2_O_2_ (**a**), pH 4.0 (**b**) or 2 M NaCl (**c**) for 30 min. Statistical analyses for the rest of the assays were performed using unpaired two-tailed Student’s *t*-test. Data represent the mean ± SEM of three biological replicates, each of which was performed with three technical replicates. ***P < 0.001

Our results indicate that TepB is a substrate of H3-T6SS, which is negatively regulated by OxyR and OmpR (Figs. 1 and 2), suggesting that the function of TepB is related to environmental cues sensed by these regulatory proteins. OxyR is a global regulator of the oxidative stress response; therefore, we investigated whether H3-T6SS and TepB play roles in protection against oxidative stress in *P. aeruginosa* PA14. We found that H_2_O_2_ tolerance was reduced in the H3-T6SS mutant Δ*icmF3* and Δ*tepB* compared with the WT strain, and that resistance was restored to WT levels through complementation of the *icmF3* and *tepB* genes (Fig. 3a). Our data indicate that H3-T6SS and TepB contribute to the survival of *P. aeruginosa* PA14 cells under oxidative stress conditions. OmpR regulates the expression of genes in response to changes in osmolarity and pH. The direct regulation of H3-T6SS by OmpR prompted us to examine whether H3-T6SS and TepB are involved in pH and osmotic stress resistance. We assessed the viability of the *P. aeruginosa* PA14 H3-T6SS mutants Δ*icmF3* and Δ*tepB* following incubation at pH 4.0 for 30 min. The Δ*icmF3* and Δ*tepB* mutants showed much lower survival rates than that of the WT after treatment at pH 4.0, and the WT survival phenotype was restored after complementation of the *icmF3* and *tepB* genes (Fig. 3b). Similar results were obtained in these strains after treatment with 2 M NaCl (Fig. 3c). Our results indicate that H3-T6SS and TepB contribute to the survival of *P. aeruginosa* PA14 under oxidative, pH, and osmotic stress conditions.

### H3-T6SS and TepB influence the motility of *P. aeruginosa* PA14

**Fig. 4:**
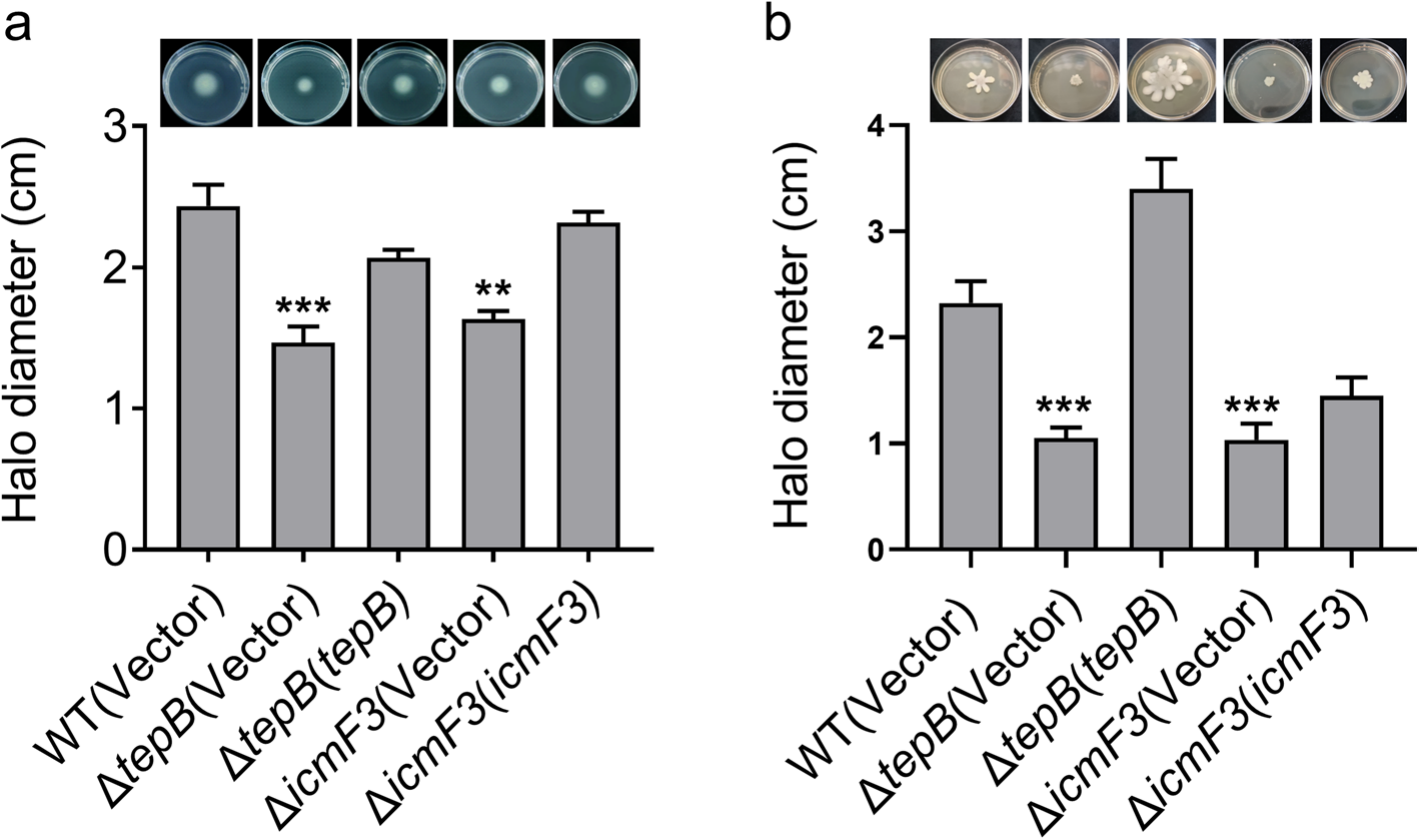
The motility is influenced by H3-T6SS and TepB. **a** The swimming motility of *P. aeruginosa* PA14 strains on motility agar plates containing 1 mM IPTG and 100 μg/mL tetracycline after 30 h of incubation at 30 °C. **b** The swarming motility of *P. aeruginosa* PA14 strains on the swarm plates containing 1 mM IPTG and 100 μg/mL tetracycline after 36 h of incubation at 30 °C. Data represent the mean ± SEM of three biological replicates, each of which was performed with three technical replicates. **P < 0.01; ***P < 0.001

For many bacteria, motility is crucial to survival, growth, biofilm formation, and virulence. Motility enables bacteria to move toward resources and supports the dispersal of progeny (Nan and Zusman 2016). Bacteria have developed several motility mechanisms to exploit available environments (Wadhwa and Berg 2021), including swimming and swarming, which are the most common motility styles (Rashid and Kornberg 2000; Burrows 2012). As T6SS is involved in motility in *Citrobacter freundii* and *Xanthomonas phaseoli* (Liu et al. 2015; Montenegro Benavides et al. 2021), we investigated whether H3-T6SS and TepB are also involved in motility in *P. aeruginosa* PA14. The mutants Δ*icmF3* and Δ*tepB* were significantly less motile than the WT strain, and this motility defect was fully restored upon complementation (Fig. 4a). We compared swarming motility among the WT, Δ*icmF3*, Δ*tepB*, and complemented Δ*icmF3*(*icmF3*) and Δ*tepB*(*tepB*) strains. Swarming motility was weaker in the Δ*icmF3* and Δ*tepB* mutants than in the WT strain, and the motile phenotype was restored in the mutants via complementation of the *icmF3* and *tepB* genes (Fig. 4b). Collectively, these results suggest that H3-T6SS and TepB influence the motility of *P. aeruginosa* PA14.

### H3-T6SS and TepB influence biofilm formation by *P. aeruginosa* PA14

**Fig. 5:**
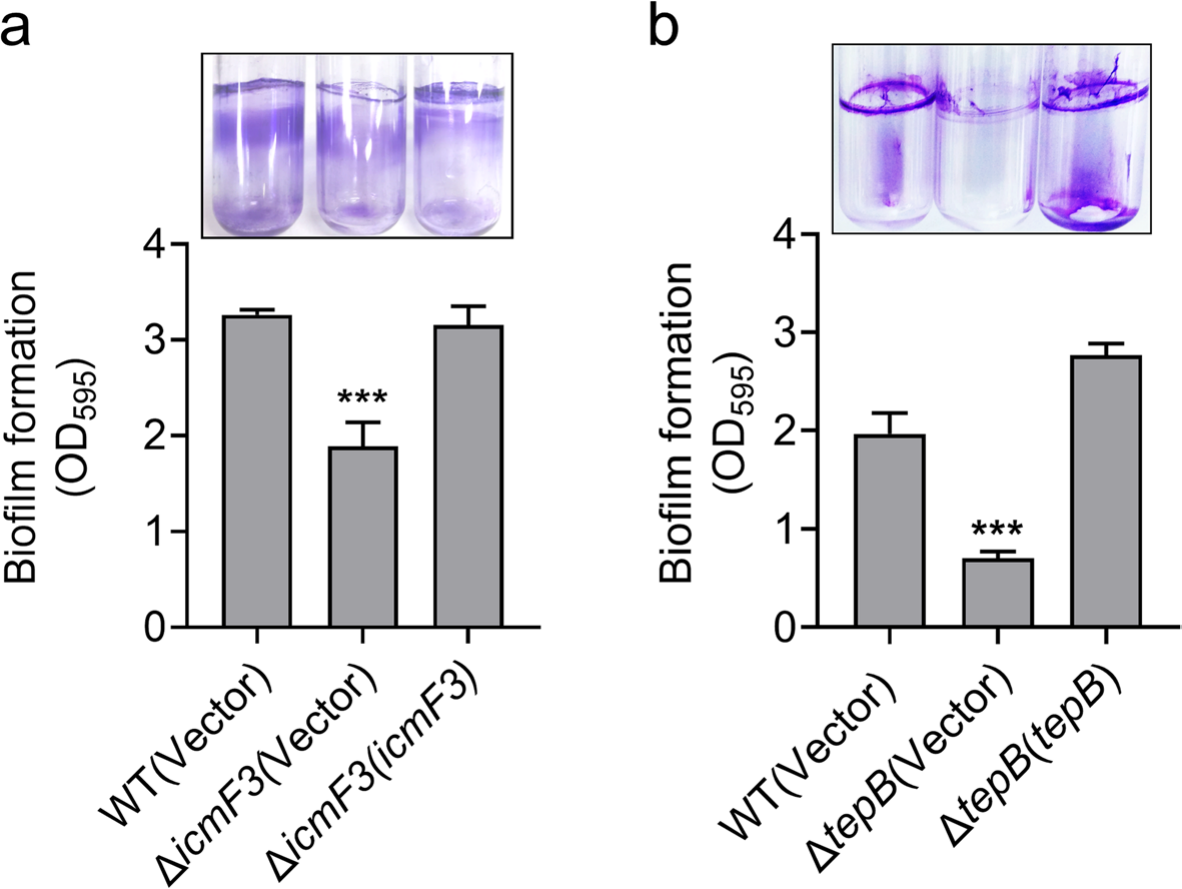
H3-T6SS and TepB influence biofilm formation. **a** and **b** Saturated bacterial cultures were diluted 100-fold in fresh TSB medium. After vertical incubation for 2 days with shaking at 120 rpm at 37 °C, biofilm formation of the strains was determined by crystal violet staining and quantified using optical density measurement. Data represent the mean ± SEM of three biological replicates, each of which was performed with three technical replicates. ***P < 0.001

Biofilm formation is governed by adaptive responses to microenvironmental cues, and involves motility (Tolker-Nielsen 2015). For the pathogen *P. aeruginosa*, biofilms represent an important virulence factor that plays a role in infection and the avoidance of host immunity (Al-Wrafy et al. 2017). Therefore, we examined the effects of H3-T6SS and TepB on biofilm formation using the crystal violet biofilm assay. We found that biofilm formation was defective in the strains Δ*icmF3* and Δ*tepB* compared with the WT (Fig. 5a and b). Biofilm formation was restored to the WT level in the mutant lines following complementation of the *icmF3* and *tepB* genes (Fig. 5a and b). These results suggest that H3-T6SS and TepB play pivotal roles in biofilm formation, and we newly identify TepB as a biofilm-promoting T6SS effector.

### TepB is involved in the virulence of *P. aeruginosa* PA14 toward *Caenorhabditis elegans*

**Fig. 6:**
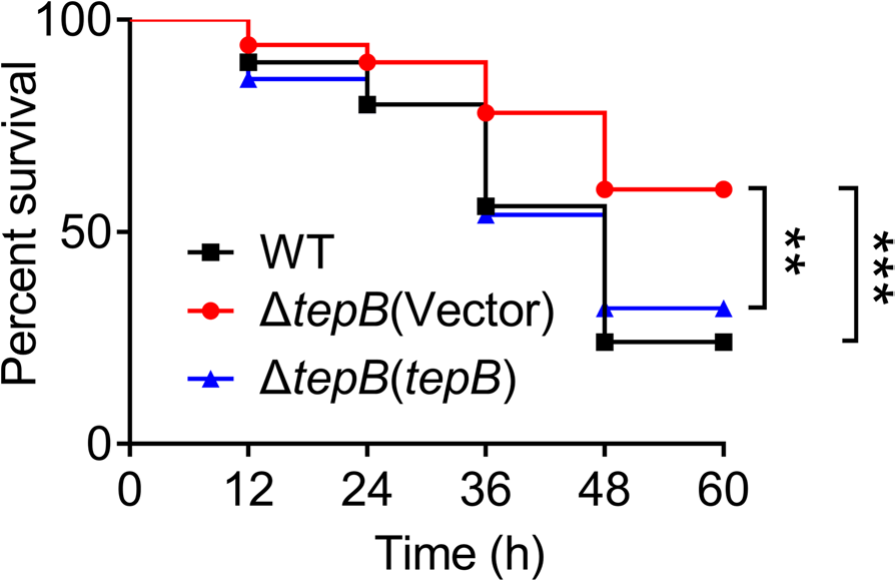
TepB is involved in virulence toward *C. elegans*. *C. elegans* were infected with *P. aeruginosa* PA14 wild-type, Δ*tepB* mutant and the complemented Δ*tepB*(*tepB*) strains. The survival rate of *C. elegans* after infection by *P. aeruginosa* PA14 was shown in 48 h. Data represent the mean of three biological replicates, each of which was performed with three technical replicates. Statistical analysis was performed by Log-Rank test. **P < 0.002; ***P < 0.001

Our results suggest that the H3-T6SS effector TepB influences motility and biofilm formation, which are crucial to virulence in *P. aeruginosa* PA14. The *P. aeruginosa* PAO1 H3-T6SS mutant strains Δ*clpV3* and Δ*icmF3* exhibited reduced virulence in a worm model (Sana et al. 2013; Lin et al. 2015). Therefore, we investigated whether the H3-T6SS effector TepB is involved in the pathogenicity of *P. aeruginosa* PA14 using a *C. elegans* infection model. The worms were infected with the WT, Δ*tepB* mutant, and complemented Δ*tepB*(*tepB*) strains. Infection with the WT strain resulted in a 27% survival rate for *C. elegans* within 48 h of infection, and the survival rate was increased to 57% after infection with the Δ*tepB* mutant, which was restored to the WT level when complemented with *tepB* gene. We also found that the survival rate of *C. elegans* was significantly higher at each time point after infection with the Δ*tepB* mutant compared with the WT and complemented Δ*tepB*(*tepB*) strains (Fig. 6). Together, these data suggest that TepB is required for virulence in *P. aeruginosa* PA14.

## Discussion

T6SS of *P. aeruginosa* plays important roles in pathogenicity to host cells and adaptation to various environments (Chen et al. 2015). Regulation of T6SS allows *P. aeruginosa* to respond to its environment (Chen et al. 2015). In *P. aeruginosa* PA14, RsmA downregulates the expression of all three T6SS loci. AmrZ regulates H2-T6SS negatively and H1- and H3-T6SS positively, via direct binding to their promoters (Allsopp et al. 2017). LasR and MvfR suppress the expression of H1-T6SS during *P. aeruginosa* pathogenesis but activate H2- and H3-T6SS (Lesic et al. 2009; Maura et al. 2016). RetS negatively controls the expression of H1- and H3-T6SS. In this study, we found that two transcriptional regulators, OxyR and OmpR, negatively regulate the expression of H3-T6SS via direct binding to its promoter. OxyR and OmpR control the expression of H3-T6SS and an effector protein involved in oxidative stress resistance, pH and osmotic stress tolerance, and biofilm formation. These findings clarify the regulation and function of H3-T6SS in *P. aeruginosa* PA14.

OxyR is a master regulator of oxidative stress in bacteria. In *P. aeruginosa*, OxyR is the most important regulator of the responses to H_2_O_2_ and organic peroxide stresses (da Cruz Nizer et al. 2021). OxyR has been reported to regulate over 100 genes involved in oxidative stress resistance, swarming, virulence, and other biological processes in *P. aeruginosa* (Vinckx et al. 2010; Wei et al. 2012; Panmanee et al. 2017). Furthermore, OxyR controls the secretion of potent cytotoxic factors in a manner partially dependent on the type III secretion system (Melstrom Jr. et al. 2007). OxyR regulation of SecA and other secreted proteins, but not T6SS proteins, has been reported previously (Wei et al. 2012; Panmanee et al. 2017). In the present study, we found that T6SS and TepB, which play roles in tolerance to pH and oxidative stresses, biofilm formation, motility, and virulence in *P. aeruginosa* PA14, were downregulated by OxyR. As a versatile bacterial weapon, T6SS secretes various cytotoxic effectors to facilitate bacterial competition and virulence (Coulthurst 2019). This study provides insights into the mechanism by which OxyR controls the secretion of cytotoxic factors.

OmpR is a global regulator of the responses to pH and osmotic stresses, and is widely distributed among bacteria (Gerken et al. 2020; Kenney and Anand 2020). OmpR regulates the expression of T6SS in *Y. pseudotuberculosis* in response to pH and osmotic stresses (Gueguen et al. 2013; Zhang et al. 2013). However, the function of OmpR in *P. aeruginosa* remains unclear. AlgR1, a homolog of OmpR, activates the expression of *algD* under osmotic stress by binding to its promoter in *P. aeruginosa* (Kato and Chakrabarty 1991). Another homolog, PhoB, is involved in crosstalk in *P. aeruginosa*, affecting bacterial behavior (Bielecki et al. 2015)*.* In this study, we identified OmpR in *P. aeruginosa* PA14 and found that it suppresses the expression of H3-T6SS. Furthermore, our results indicate that H3-T6SS and its effector protein are involved in acid and osmotic stress resistance. Our results are consistent with the reported functions of OmpR in other bacteria (Gueguen et al. 2013; Zhang et al. 2013).

The *tepB* gene is located within the *H3-T6SS* gene cluster and in the same operon as *clpV3* and *vgrG3* (Fig. 2a). *tepB* was predicted to encode a T6SS effector and to contribute to the virulence of *P. aeruginosa* PA14 (Lesic et al. 2009). In this study, we demonstrated that TepB interacts with VgrG3 and translocates into host cells (Fig. 2c and d). Proteins encoded by genes in close proximity to *vgrG* and that interact with VgrG are typically effectors secreted by T6SS (Hachani et al. 2014; Wettstadt 2020; Wu et al. 2020). Therefore, TepB was considered a T6SS effector. T6SS secretes effectors in two ways: transporting the substrate into the environment or injecting the effector into other bacteria or host cells via direct contact (Lin et al. 2021). We detected no TepB in the supernatant but observed it in host cells (Fig. 2d), suggesting that TepB is an injected effector. TepB may be secreted by T6SS in a contact-dependent manner as reported previously. We found that TepB is also involved in motility, biofilm formation, stress tolerance, and pathogenicity in *P. aeruginosa* PA14. TepB is absent in *P. aeruginosa* PAO1, which is less virulent than *P. aeruginosa* PA14. This effector may contribute to the superior virulence of *P. aeruginosa* PA14 compared with *P. aeruginosa* PAO1, similar to the function of the H2-T6SS effector PldA in the virulence of clinically isolated infectious *P. aeruginosa* (Boulant et al. 2018). Although we revealed the primary functions of TepB in this work, the molecular mechanisms underlying these functions require further investigation.

T6SS has versatile functions in stress resistance, biofilm formation, metal acquisition, and pathogenicity (Lin et al. 2017, 2021; Coulthurst 2019). The functions of H3-T6SS in *P. aeruginosa* PA14 include tolerance to H_2_O_2_, acid and osmotic stresses, motility, biofilm formation, and pathogenicity, consistent with reported T6SS functions in other bacterial species. The involvement of T6SS in oxidative and acid stress resistance has not been reported previously in *P. aeruginosa.* The pathogens *P. aeruginosa, Mycobacterium tuberculosis,* and *Yersinia pestis* form biofilms, enhancing their ability to survive and defend themselves within a host rather than as individual planktonic cells (Darby et al. 2002; Kumar et al. 2017). Multiple factors including regulatory proteins, membrane proteins, secretion systems, motility, and attachment, as well as environmental conditions such as temperature, affect biofilm formation in *P. aeruginosa* during persistent infections (Whiteley et al. 2001; Kim et al. 2020). The expression levels of the three T6SS types are relatively high in *P. aeruginosa* PAO1 biofilm cells. H1-T6SS is not involved in biofilm formation, but it affects swarming (Chen et al. 2020). IcmF3, a component of H3-T6SS, decreases biofilm formation, and increases swarming in *P. aeruginosa* PAO1 (Lin et al. 2015). We found that H3-T6SS and its effector TepB increases swimming, swarming, and biofilm formation in *P. aeruginosa* PA14. H3-T6SS plays a different role in biofilm formation in *P. aeruginosa* PA14 than that in *P. aeruginosa* PAO1 (Lin et al. 2015), and this may be responsible for the difference in biofilm invasion strategies between these two strains (Kasetty et al. 2021). Motility and biofilm formation contribute to acute and chronic infections, respectively (Balasubramanian et al. 2013). Our results suggest that H3-T6SS and TepB play roles in both acute and chronic *P. aeruginosa* PA14 infections.

TepB is a newly identified biofilm-promoting protease effector secreted by T6SS. Extracellular enzymes, such as polysaccharide-degrading hydrolases, esterases, nucleases, proteases, and lyases, are crucial for matrix turnover during biofilm formation, detachment, and dispersal (Flemming et al. 2022). For example, the serine protease autotransporter family protein SepA can promote biofilm formation by processing Aap and AtlE extracellularly in *Staphylococcus epidermidis* (Martinez-Garcia et al. 2018). The PAO1 extracellular elastase LasB has been reported to promote biofilm formation partly via rhamnolipid-mediated regulation (Yu et al. 2014). PAO1 secretes the DNA-specific endonuclease EndA to degrade extracellular DNA in biofilms, leading to the dispersal of PAO1 from the biofilm (Cherny and Sauer 2019). Membrane proteins and secretion systems, including TolA, OmlA, the twin arginine translocation pathway, type II secretion system, type III secretion system, and T6SS, are involved in the translocation of the biofilm matrix and extracellular enzymes (Whiteley et al. 2001; Lin et al. 2021; Flemming et al. 2022). However, few effectors associated with secretion systems, especially in T6SS, have been found to promote biofilm formation. Only VgrG and Hcp, which are both components and effectors of the T6SS, have been found to play positive roles in biofilm formation in bacteria (Sha et al. 2013; Fei et al. 2022; Pan et al. 2022). TepB is the first identified enzymatic effector of T6SS that promotes biofilm formation during acute and chronic infections of *P. aeruginosa* PA14. The mechanism by which TepB promotes biofilm formation via metalloprotease activity requires further study.

In conclusion, we found that the transcriptional regulators OxyR and OmpR downregulate the expression of H3-T6SS in pathogenic *P. aeruginosa* PA14 via direct binding to its promoter region. H3-T6SS and its biofilm-promoting effector TepB improve tolerance to oxidative, acid, and osmotic stresses, motility, biofilm formation, and virulence in *P. aeruginosa* PA14. This study elucidated the regulation and functions of H3-T6SS in *P. aeruginosa* PA14, although details of the underlying mechanisms requires further investigation.

## Materials and methods

### Bacterial strains and growth conditions

Bacterial strains and plasmids used in this study are listed in Supplementary Table S1. *Escherichia coli* strains were grown at 37 °C in either Luria-Bertani (LB) broth or agar. *P. aeruginosa* PA14 strains were grown at 37 °C in tryptic soy broth (TSB) medium or M9 minimal medium (6 g/L Na_2_HPO_4_, 3 g/L KH_2_PO_4_, 0.5 g/L NaCl, 1 g/L NH_4_Cl, 2 mM MgSO_4_, 0.1 mM CaCl_2_, 0.2% glucose, pH 7.0). The *P. aeruginosa* PA14 strain was the parent of all derivatives used in this study. To generate in-frame deletion mutants, the pK18*mobsacB* derivatives were transformed into relevant *P. aeruginosa* PA14 strains through *E. coli* S17–1-mediated conjugation and screened as described previously (Lin et al. 2017; Li et al. 2021). Antibiotics were used at the following concentrations for *E. coli*: kanamycin, 50 μg/mL; tetracycline, 15 μg/mL; gentamicin, 10 μg/mL; and for *P. aeruginosa* PA14: kanamycin, 50 μg/mL; chloramphenicol, 30 μg/mL; gentamicin, 200 μg/mL; tetracycline, 200 μg/mL for plates or 160 μg/mL for liquid growth. All chemicals were of Analytical Reagent Grade purity or higher.

### Plasmid construction

Primers used in this study are listed in Supplementary Table S2. The plasmid pK18-Gm-Δ*tepB* (*PA14_33970*) was used to construct the Δ*tepB* in-frame deletion mutant of *P. aeruginosa* PA14. A 679-bp upstream fragment and an 866-bp downstream fragment of *tepB* were amplified using the primer pairs *PA14_33970*-Up-F-*Bam*HI/*PA14_33970*-Up-R and *PA14_33970*-Down-F/*PA14_33970*-Down-R-*Hind*III, respectively. The upstream and downstream PCR fragments were ligated by overlapping PCR, and the resulting PCR product was digested with *Bam*HI/*Hind*III and inserted into the *Bam*HI/*Hind*III sites of the suicide vector pK18-Gm to produce pK18-Gm-Δ*tepB*. The knock-out plasmids pK18-Gm*-icmF3* (*PA14_34130*), pK18-Gm-*oxyR* (*PA14_70560*) and pK18-Gm-*ompR* (*PA14_68700*) were constructed in a similar manner by using primers list in Supplementary Table S2. To complement the Δ*tepB* mutant, primers *PA14_33970*-F-*Eco*RI/*PA14_33970*-R-*Bgl*II were used to amplify the *tepB* gene from the *P. aeruginosa* PA14 genome DNA. The PCR product of *tepB* was digested with *Eco*RI/*Bgl*II and cloned into the *Eco*RI/*Bgl*II sites of plasmid pME6032 to produce pME6032-*tepB*. The complementation plasmids pME6032-*icmF3,* pME6032-*oxyR,* and pME6032-*ompR* were similarly constructed by using primers list in Supplementary Table S2. To construct pME6032-*tepB*-*vsvg*, primers *PA14_33970-*F-*Eco*RI/*PA14_33970*-R-*vsvg*-*Bgl*II was used to amplify the *tepB* gene and the PCR product was digested with *Eco*RI/*Bgl*II and cloned into similarly digested pME6032 to generate pME6032-*tepB*-VSVG. The plasmid pME6032-*vgrG3*-*vsvg* was constructed in a similar method by using primers list in Supplementary Table S2. For constructing expression plasmids, the genes encoding *P. aeruginosa* TepB, PA14_33980, OxyR and OmpR were amplified by PCR. The obtained DNA fragments were digested and inserted into similar digested pGEX-6p-1 and pET28a, yielding corresponding plasmids, respectively. For complementation, complementary plasmids pME6032-*oxyR*, pME6032-*ompR*, pME6032-*icmF3* and pME6032-*tepB* were introduced into respective mutants by electroporation. The integrity of the insert in all constructs was confirmed by DNA sequencing.

### Purification of recombinant proteins and Western blotting

His_6_- and GST-tagged recombinant proteins were expressed and purified from *E. coli* as describe (Shen et al. 2009). In short, the pET28a and pGEX-6p-1 derivatives were transformed into BL21(DE3) and XL1-Blue host strains, respectively. Bacteria were cultured at 37 °C in LB medium to an OD_600_ of 0.6, shifted to 18 °C and induced with 0.5 mM IPTG for 16 h. Harvested cells were disrupted by sonication, and His_6_- or GST-tagged proteins were purified with the His•Bind Ni-NTA resin (Novagen, Madison, WI) or the GST•Bind resin (Novagen, Madison, WI) according to manufacturer’s instructions. Purified recombinant proteins were dialyzed against the appropriate buffer overnight at 4 °C and stored at − 80 °C until use. Protein concentrations were measured using the Bradford assay according to the manufacturer’s instructions (Bio-Rad, Hercules, CA) with bovine serum albumin as standard.

For Western blotting, samples resolved by SDS–PAGE were transferred onto polyvinylidene difluoride membranes. After blocking with 5% (w/v) BSA in TBST buffer (50 mM Tris pH 7.4, 150 mM NaCl, 0.05% Tween 20), membranes were incubated with the appropriate primary antibody: anti-VSVG (Santa Cruz Biotechnology, USA), 1:5000 and anti-GST (Zhongshan Golden Bridge Biotechnology, Beijing, China), 1:2000. The membrane was washed three times in TBST buffer and incubated with 1:10,000 dilution of horseradish peroxidase-conjugated secondary antibodies (Shanghai Genomics, Shanghai, China) for 2 h. Signals were detected using the ECL plus kit following the manufacturer’s specified protocol.

### Electrophoretic mobility shift assay (EMSA)

Electrophoretic mobility shift assay was performed as previously described (Si et al. 2017a). Briefly, Bio-P_H3-T6SS left_ was amplified from the *P. aeruginosa* PA14 genome DNA with primers P_H3-T6SS left_-F/P_H3-T6SS left_-R (labeled with biotin). The unlabeled P_H3-T6SS left_ competitor probe was amplified from the *P. aeruginosa* PA14 genome DNA with primers P_H3-T6SS left_-F/P_H3-T6SS left_-R. All PCR products were purified by EasyPure Quick Gel Extraction Kit (TransGen Biotech, Beijing, China). Each 20-μL EMSA reaction solution was prepared by adding the following components according to the manufacturer’s protocol (LightShift Chemiluminescent EMSA Kit, Thermo Fisher Scientific, CA, USA): 1× binding buffer, 2.5% glycerol, 5 mM MgCl_2_, 0.05% NP-40, 5 mM EDTA, 20 ng probe and 0–0.5 ng protein. Reaction solutions were incubated for 20 min at 26 °C. The protein-probes mixtures were separated by using a 6% polyacrylamide native gel and transferred to a Biodyne B Nylon membrane (Thermo Fisher Scientific, CA, USA). Migration of biotin-labeled probe was detected by streptavidin-horseradish peroxidase conjugates that bind to biotin and chemiluminescent substrate according to the manufacture’s protocol.

### Construction of chromosomal fusion reporters and β-galactosidase assays

The *lacZ* fusion reporter vector pMini-CTX-*P*_*H3-T6SS left*_*::lacZ* and pMini-CTX-*P*_*H3-T6SS3 right*_*::lacZ* were transformed into *E. coli* S17–1 *λpir* and mated with *P. aeruginosa* PA14 strains as described previously (Hoang et al. 2000). Promoter fragments were integrated at the CTX phage attachment site (*attB*) in strain *P. aeruginosa* PA14 and the interrelated mutant strains, and the pFLP2 plasmid expressing Flp recombinase was used to excision of the Tc^r^ marker following the protocol to obtain the unmarked transcriptional fusion strains (Hoang et al. 2000). The *lacZ* fusion reporter strains were grown in TSB medium with or without 1 mM H_2_O_2_ at 37 °C. The β-galactosidase activity was assayed using ONPG (o-Nitrophenyl β-D-galactopyranoside) as the substrate and expressed in Miller units.

### Analysis of cotranscription by reverse transcription-PCR (RT-PCR)

Gene cotranscription assay was performed as previously described (Zheng et al. 2014). Mid-exponential phase *P. aeruginosa* PA14 strains grown in M9 medium were harvested and RNA was extracted using the *SteadyPure* Universal RNA Extraction Kit AG21017 (Accurate Biotechnology, Hunan, China), and treated with RNase-free DNase I according to the manufacturer’s protocol after its integrity was checked by agarose electrophoresis. First-strand cDNA was reverse transcribed from 1 μg DNase I-digested RNA using the *Evo M-MLV* RT Kit with gDNA Clean for qPCR AG11705 (Accurate Biotechnology, Hunan, China) according to the manufacturer’s protocol. The resulting cDNA was used as the template to amplify the intragenic regions of *PA14_33990*, *tepB* and *PA14_33960* genes with 2 × Accurate Master Mix AG1107 (Accurate Biotechnology, Hunan, China). ddH_2_O and No Reverse Transcriptase (NRT) sample were used as negative controls, respectively. The specific primers used for amplification are list in Supplementary Table S2.

### GST pull-down assay

The GST pull-down assay was performed as previously described with minor modifications (Xu et al. 2010). To verify the interaction between TepB with VgrG3, stationary phase *P. aeruginosa* PA14 cells expressing VgrG3-VSVG protein were lysed in Bugbuster solution (Novagen, Madison, WI). Cleared cell lysates were incubated with 10 μg purified GST-TepB on a rotator at 4 °C overnight, and 40 μL prewashed glutathione-Sepharose beads (Novagen, Madison, WI) were added to the reactions. After another 4 h of incubation at 4 °C, the beads were washed six times with TEN buffer (100 mM Tris pH 8.0, 10 mM EDTA, 500 mM NaCl). Retained proteins were detected by immunoblotting after SDS–PAGE.

### Bacterial survival assay

Mid-logarithmic phase *P. aeruginosa* strains grown in TSB medium were collected, washed and diluted 100-fold into M9 medium, and then treated with or without H_2_O_2_ (1.0 mM), 2 M NaCl or pH 4.0 for 30 min at 37 °C. After treatment, the cultures were serially diluted and plated onto LB agar plates, and colonies were counted after 24 h growth at 37 °C. Percentage survival was calculated by dividing the CFU number of stressed cells by the CFU number of unstressed cells (Song et al. 2015). All these assays were performed in triplicate at least three times.

### Motility assay

Swimming motility assay was performed as previously described (Inoue et al. 2008; Li et al. 2019). Briefly, 1 μL bacterium solution was injected into semi-solid agar medium (1% tryptone, 0.5% NaCl, 0.3% Difco Bacto agar) and incubated for 30 h under 30 °C before observation. Motility halos were measured after 30 h of incubation. The swarming motility assay was performed as previously described (Rashid and Kornberg 2000). Briefly, a single colony selected from TSB plates was touched slightly on soft agar medium (8 g/L Nutrient Broth, 5 g/L glucose, 0.5% Difco Bacto agar) and incubated for 36 h under 30 °C before observation, and then motility halos were measured.

### Biofilm formation assay

Biofilm formation was determined following the methods of O’Toole and Zhang (O’Toole and Kolter 1998; Zhang et al. 2020). Briefly, overnight bacterial cultures were diluted 100-fold in fresh 4 mL TSB medium with appropriate antibiotics when necessary. After vertical incubation for 2 days with the shake of 120 rpm at 37 °C, the bacterial cultures were removed and the test tubes were washed twice with fresh phosphate buffered saline (PBS). The cells that adhered to the tubes were stained with 0.1% crystal violet for 30 min and then washed twice with PBS. The cell-bound dye was dissolved in 5 mL of 95% ethanol, and the absorbance of the eluted solution was measured using a microplate reader at 595 nm.

### *Caenorhabditis elegans* killing assay

*P. aeruginosa* strains were grown overnight at 37 °C and supplemented with nematode growth medium (NGM) following the published method (Tan et al. 1999). The NGM plates were incubated firstly at 37 °C for 24 h and then at 25 °C for 24 h before seeding with adult hermaphrodite worms. Before solidification, all experimental plates were added to 200 μM 5-fluorodeoxyuridine, which was used to prevent the development of progeny. In each assay, 40–50 adult nematodes were transferred to a single plate. Plates were incubated at 25 °C and scored for live worms every 12 h for total time of 48 h. The experiments were conducted in triplicate and *E. coli* OP50 was used as the negative control. A worm was considered dead when it no longer responded to touch. Any worms that died as a result of getting stuck to the wall of the plate were excluded from the analyses.

### Translocation assay for TEM1 fusion protein

The translocation assay for TEM1-TepB fusion protein was performed as previously described (Jiang et al. 2014; Zhu et al. 2021). HeLa cells were grown in 96-well black-wall, clear-bottom plates and infected with PA14 WT or T6SS deficient mutant strains with TEM1-TepB (at an MOI of 100) for 3 h. Host cells were then washed with PBS for three times and treated with CCF2-AM (LiveBLAzer FRET-B/G Loading Kit, Invitrogen) for 90 min at room temperature. Samples were examined with a Nikon fluorescence microscope (Nikon, Japan).

### Statistical analysis

All experiments were performed at least in triplicate and repeated on two different occasions. Data are expressed as mean ± SEM. Differences between frequencies were assessed by the Student’s *t*-test (bilateral and unpaired). Statistical analysis of results was conducted by using GraphPad Prism 8 (GraphPad Software, San Diego California, USA), using a *P* value of < 0.05 as statistically significant. The survival times of *Caenorhabditis elegans* were analyzed using Kaplan-Meyer curves and the comparisons were performed using the Log-Rank test. *P* value < 0.033 was used as statistically significant.

## Supplementary Information

**Additional file 1: Fig. S1.** Promoter activity analysis under oxidative stress. **Table S1.** Bacterial strains and plasmids used in this study. **Table S2.** Primers used in this study. Supplementary References.

## Data Availability

All datasets generated for this study are included in the article/Supplementary Information.
