# Supplementary material for: H3-T6SS of Pseudomonas aeruginosa PA14 contributes to environmental adaptation via secretion of a biofilm-promoting effector
Source: Stress Biol. 2022 Dec 28;2(1):55. doi: 10.1007/s44154-022-00078-7 (PMC10442045; doi:10.1007/s44154-022-00078-7)
Supplement: Supplementary file 1 — Additional file 1: Fig. S1. Promoter activity analysis under oxidative stress. Table S1. Bacterial strains and plasmids used in this study. Table S2. Primers used in this study. Supplementary References. [file 44154_2022_78_MOESM1_ESM.docx]

**Supplementary Information**

**H3-T6SS of *Pseudomonas aeruginosa* PA14 contributes to environmental adaptation via secretion of a biofilm-promoting effector**

**This PDF file includes:**

**Supplementary Figure 1**

**Supplementary Tables 1-2**

**Supplementary References**

# Supplementary Figure


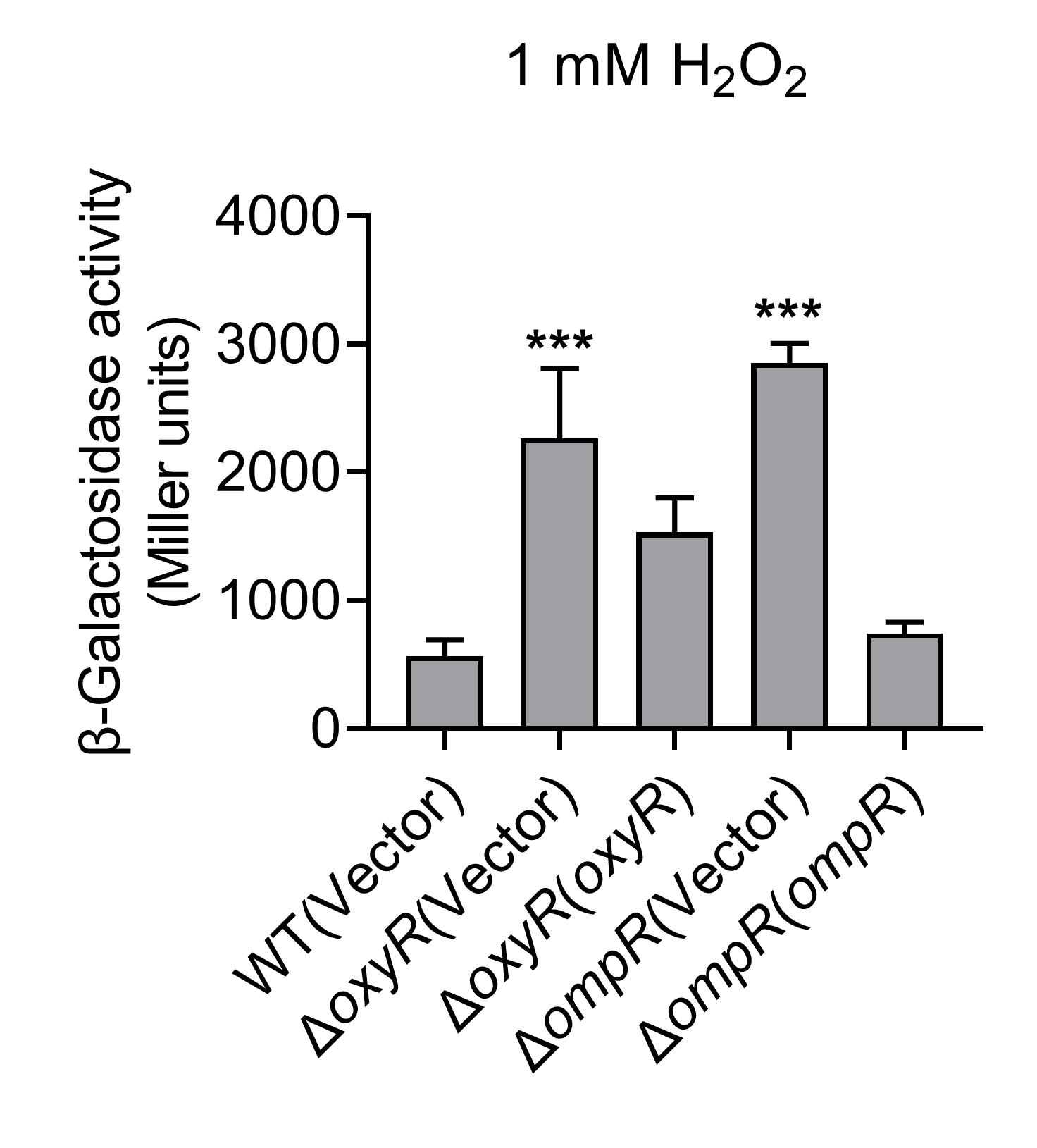


**Fig.S1 Promoter activity analysis under oxidative stress.** β-galactosidase analyses of H3-T6SS promoter activities by using the transcriptional *P_H3-T6SS_::lacZ* chromosomal fusion reporter expressed in the *P. aeruginosa* PA14 strains grown to stationary phase in TSB medium with 1 mM H_2_O_2_. Data represent the mean ± SEM of three biological replicates, each of which was performed with three technical replicates. ****P*< 0.001.

# Supplementary Tables

**Table S1. Bacterial strains and plasmids used in this study.**

| **Strain or plasmid** | **Relevant characteristics** | **Reference** |
| --- | --- | --- |
| ***E. coli*** |  |  |
| S17-1*λ pir* | *λ*-*pir* lysogen of S17-1, *thi pro hsdR hsdM^+^ recA* RP4-Tc::Mu-Km::Tn7 | Laboratory stock |
| BL21(DE3) | Host for expression vector pET28a | Novagen |
| XL-1 Blue | Host for expression vector pGEX-6p-1 | Novagen |
| DH5α | FΦ80Δ*lacZ*ΔM15/Δ(*lacZYA-argF*)*U169recA1 endA1 hsdR17* | Laboratory stock |
| ***P. aeruginosa*** |  |  |
| PA14 | Wild-type *Pseudomonas aeruginosa*, Km^r^ | Laboratory stock |
| Δ*oxyR* | *oxyR* deletion *mutant in* PA14, Km^r^ | This study |
| Δ*ompR* | *ompR* deletion mutant in PA14, Km^r^ | This study |
| Δ*tepB* | *tepB* deletion mutant in PA14, Km^r^ | This study |
| WT(Vector) | PA14 wild type containing pME6032, Km^r^, Tc^r^ | This study |
| Δ*tepB*(Vector) | Δ*tepB* containing pME6032, Km^r^, Tc^r^ | This study |
| Δ*tepB*(*tepB*) | Δ*tepB* containing pME6032-*tepB*, Km^r^, Tc^r^ | This study |
| Δ*icmF3*(Vector) | Δ*icmF3* containing pME6032, Km^r^, Tc^r^ | This study |
| Δ*icmF3*(*icmF3*) | Δ*icmF3* containing pME6032-*icmF3*, Km^r^, Tc^r^ | This study |
| **Plasmids** |  |  |
| pK18*mobsacB* | *sacB*-based gene replacement vector, Km^r^ | ([Schafer et al. 1994](#_ENREF_6)) |
| pK18-Gm | *pK18mobsacB* carrying Gm coding region, Km^r^, Gm^r^ | ([Li et al. 2021](#_ENREF_5)) |
| pK18-Gm-Δ*oxyR* | Construct used for in-frame deletion of *oxyR*, Km^r^, Gm^r^ | This study |
| pK18-Gm-Δ*ompR* | Construct used for in-frame deletion of *ompR*, Km^r^, Gm^r^ | This study |
| pK18-Gm-Δ*icmF3* | Construct used for in-frame deletion of *icmF3*, Km^r^, Gm^r^ | This study |
| pK18-Gm-Δ*tepB* | Construct used for in-frame deletion of *tepB*, Km^r^, Gm^r^ | This study |
| pFLP2 | Source of Flp recombinase, Amp^r^ | ([Hoang et al. 1998](#_ENREF_3)) |
| pMini-CTX::*lacZ* | Ω-*FRT*-*attP*-MCS, *ori*, *int*, *oriT*, Tc^r^ | ([Becher and Schweizer 2000](#_ENREF_1); [Hoang et al. 2000](#_ENREF_4)) |
| pMini-CTX-*P_H3-T6SS left_::lacZ* | Mini-CTX::*lacZ* containing H3-T6SS promoter, Tc^r^ | This study |
| pMini-CTX-*P_H3-T6SS right_::lacZ* | Mini-CTX::*lacZ* containing H3-T6SS promoter, Tc^r^ | This study |
| pME6032 | Shuttle vector containing *lacI*^q^-*Ptac* fragment for gene expression, Tc^r^ | ([Heeb et al. 2002](#_ENREF_2)) |
| pME6032-*oxyR* | *oxyR* cloned into pME6032 for complementation, Tc^r^ | This study |
| pME6032-*ompR* | *ompR* cloned into pME6032 for complementation, Tc^r^ | This study |
| pME6032-*icmF3* | *icmF3* cloned into pME6032 for complementation, Tc^r^ | This study |
| pME6032-*tepB* | *tepB* cloned into pME6032 for complementation, Tc^r^ | This study |
| pME6032-*tepB-vsvg* | pME6032 expressing *tepB-vsvg*, Tc^r^ | This study |
| pME6032-*vgrG3-vsvg* | pME6032 expressing *vgrG3-vsvg*, Tc^r^ | This study |
| pET28a | Expression vector with N-terminal hexahistidine affinity tag, Km^r^ | Novagen |
| pET28a-*oxyR* | pET28a carrying *oxyR* coding region of, Km^r^ | This study |
| pET28a-*ompR* | pET28a carrying *ompR* coding region of, Km^r^ | This study |
| pGEX6p-1 | Expression vector with N-terminal GST tag, Amp^r^ | Novagen |
| pGEX6p-1-*tepB* | pGEX6p-1 carrying *tepB* coding region, Amp^r^ | This study |
| pGEX6p-1-*tepB* | pGEX6p-1 carrying *tepB* coding region, Amp^r^ | This study |
| pBBR1MCS5-TEM1-*tepB* | pBBR1MCS5 carrying *TEM1-tepB* coding region, Gm^r^ | This study |
| *Tc^r^, Gm^r^, Km^r^ and Apm^r^ represent resistance to tetracycline, gentamicin, kanamycin and ampicillin, respectively. | | |

**Table S2. Primers used in this study.**

| **Primers** | **5’-3’ sequence** | **Function** |
| --- | --- | --- |
| *oxyR*-Up-F-*Eco*RI | ACCGGAATTCCTGGTTGAGCAGCCATTCCC | To generate  pK18-Gm-Δ*oxyR* |
| *oxyR*-Up-R | TCCTGGGCGAGGGTGACGAT |  |
| *oxyR*-Down-F | ATCGTCACCCTCGCCCAGGAGAACAACCGCAAATCGCATGACC |  |
| *oxyR*-Down-R-*Hind*III | ACCCAAGCTTGGAAGCCGAGGTTGGCAAGGAA |  |
| *ompR*-Up-F-*Bam*HI | ACGCGGATCCGTCAGGGTCGCTTCGTTCTTCAT | To generate  pK18-Gm-Δ*ompR* |
| *ompR*-Up-R | GCAGGGTTCGACATAGAAACTCCC |  |
| *ompR*-Down-F | GGGAGTTTCTATGTCGAACCCTGCCGGTCTGGGGTGTCGGCTAC |  |
| *ompR*-Down-R-*Pst*I | AAAACTGCAGGCAGCAACTCCAGCGACAGG |  |
| *icmF3*-Up-F | CGTGTCTCTAGAGATGGTCGAGCGCGCCCTCGAACTG | To generate  pK18-Gm-Δ*icmF3* |
| *icmF3*-Up-R | CAGGACCAGCGGCAGCAGGCGCCAGGTAGCGCACAAC |  |
| *icmF3*-Down-F | GGCGCCTGCTGCCGCTGGTCCTGCAACTGCCGGTG |  |
| *icmF3*-Down-R | CACGACAAGCTTCGCGCGCAACGCTTCGTCGAGCTTC |  |
| *PA14_33970*-Up-F-*Bam*HI | CGCGGATCCGAAAAGCCATAGACATCCCGACCCT | To generate  pK18-Gm-Δ*tepB* |
| *PA14_33970-*Up-R | GGCGGAGGGATACGGGCTTT |  |
| *PA14_33970-*Down-F | AAAGCCCGTATCCCTCCGCCATGTAGTCGTCGCCGCTGTCC |  |
| *PA14_33970-*Down-R-*Hind*III | CCCAAGCTTCCGTCGATGCGTTGCTTGGT |  |
| *oxyR*-F-*Bam*HI | CGCGGATCCATGACCCTCACCGAACTGCG | To generate  pET28a-*oxyR* |
| *oxyR*-R-*Hind*III | CCCAAGCTTTCATGCGATTTGCGGTTGTT |  |
| *oxyR*-F-*Eco*RI | CCGGAATTCATGACCCTCACCGAACTGCGCTAC | To generate  pME6032-*oxyR* |
| *oxyR*-R-*Bam*HI | CGCGGATCCTCATGCGATTTGCGGTTGTTCCT |  |
| *ompR*-F-*Eco*RI | CCGGAATTCATGTCGAACCCTGCCGCCCTG | To generate pET28a-*ompR*, pME6032-*ompR* |
| *ompR*-R-*Xho*I | CCGCTCGAGTCAGGCCTTGCGCGCGTTG |  |
| *icmF3*-F-*Eco*RI | CCGGAATTCATGAGCGGCGCGACGCTGTTCA | To generate  pME6032-*icmF3* |
| *icmF3*-R-*Bgl*II | GGAAGATCTTCATGGGATACCTCCGGTGCTG |  |
| *PA14_33970-*F-*Eco*RI | CCGGAATTCATGCCCCGAACCATCGAA | To generate  pME6032-*tepB* |
| *PA14_33970-*R-*Bgl*II | GGAAGATCTCTACATGTAACTGATGCGTTTGCC |  |
| *PA14_33970*-F-*Bam*HI | CGCGGATCCATGCCCCGAACCATCGAATACTG | To generate  pGEX6p-1-*tepB* |
| *PA14_33970*-R-*Xho*I | CCGCTCGAGCTACATGTAACTGATGCGTTTGCCGA |  |
| *PA14_33980*-F-*Bam*HI | CGCGGATCCATGAACTGGAACGATCTTTGCGGAA | To generate  pGEX6p-1-*PA14_33980* |
| *PA14_33980*-R-*Xho*I | CCGCTCGAGTCAGCGTCGGTAGAACACCAGCC |  |
| *vgrG3*-F-*Kpn*I | CGGGGTACCATGCCCCGTCCCACCGATAGC | To generate  pME6032-*vgrG3*-VSVG |
| *vgrG3-*F-VSVG-R-*Xho*I | CCGCTCGAGTCATTTTCCTAATCTATTCATTTCAATATCTGTATAGTTGACCTTTACCAGACCGCC |  |
| *PA14_33970*-F-*Eco*RI  *PA14_33970*-R-VSVG-*Bgl*II | CCGGAATTCATGCCCCGAACCATCGAA  GGAAGATCTCTATTTTCCTAATCTATTCATTTCAATATCTGTATACATGTAACTGATGCGTTTGCC | To generate  pME6032-*tepB*-VSVG |
| P_H3-T6SS left_-F-*Eco*RI | CCGGAATTCTATGGTGAAACATCCGTGCTTCATGG | To generate  pMini-CTX-*P_H3-T6SS left_::lacZ* |
| P_H3-T6SS left_-R-*Bam*HI | CGCGGATCCGGCGGCTGACTCCGATGCAAT |  |
| P_H3-T6SS left_-F | GGGAGTCCAACGAAAATTT | For EMSA |
| P_H3-T6SS left_-R | GGCGGCTGACTCCGATGC |  |
| Control-F | ATGCCCCGAACCATCGAAT |  |
| Control-R | CGATGCGCCCGTCGGCGT |  |
| *PA14_33960*-Co-F | CCGATAGCAATACCAGCCTCTC | For RT-PCR |
| *PA14_33960*-Co-R | GGTGAGATGGGTGGCGATTG |  |
| *PA14_33970*-Co-F | ATGCCCCGAACCATCGAATACTG |  |
| *PA14_33970*-Co-R | TGTAGTTCAGCGCGCGATACAC |  |
| *PA14_33990*-Co-F | CCTGCAACGCACCCATCATTAC |  |
| *PA14_33990*-Co-R | GGTATTGCCGGCCTTGAACAG |  |
| *TEM1*-F-*Xho*I | CCGCTCGAGGATGAGTATTCAACATTTCCGTGT | To generate pBBR1MCS5-TEM1-*tepB* |
| *TEM1*-R-*Hind*III | GGGAAGCTTCCAATGCTTAATCAGTGAGG |  |
| *33970*-F-*Hind*III | CCCAAGCTTATGCCCCGAACCATCGAATAC |  |
| *33970*-R-*Eco*RI | CGAATTCCTACATGTAACTGATGCGTTTGCC |  |

* Underlined sites Indicate restriction enzyme cutting sites added for cloning.

# Supplementary References

Becher, A., and Schweizer, H.P. (2000) Integration-proficient *Pseudomonas aeruginosa* vectors for isolation of single-copy chromosomal *lacZ* and *lux* gene fusions. Biotechniques 29: 948-950, 952. https://doi:10.2144/00295bm04

Heeb, S., Blumer, C., and Haas, D. (2002) Regulatory RNA as mediator in GacA/RsmA-dependent global control of exoproduct formation in *Pseudomonas fluorescens* CHA0. J Bacteriol 184: 1046-1056. https://doi:10.1128/jb.184.4.1046-1056.2002

Hoang, T.T., Karkhoff-Schweizer, R.R., Kutchma, A.J., and Schweizer, H.P. (1998) A broad-host-range Flp-FRT recombination system for site-specific excision of chromosomally-located DNA sequences: application for isolation of unmarked *Pseudomonas aeruginosa* mutants. Gene 212: 77-86. https://doi:10.1016/s0378-1119(98)00130-9

Hoang, T.T., Kutchma, A.J., Becher, A., and Schweizer, H.P. (2000) Integration-proficient plasmids for *Pseudomonas aeruginosa*: site-specific integration and use for engineering of reporter and expression strains. Plasmid 43: 59-72. https://doi:10.1006/plas.1999.1441

Li, C., Zhu, L., Wang, D., Wei, Z., Hao, X., Wang, Z. et al. (2021) T6SS secretes an LPS-binding effector to recruit OMVs for exploitative competition and horizontal gene transfer. ISME J. https://doi:10.1038/s41396-021-01093-8

Schafer, A., Tauch, A., Jager, W., Kalinowski, J., Thierbach, G., and Puhler, A. (1994) Small mobilizable multi-purpose cloning vectors derived from the *Escherichia coli* plasmids pK18 and pK19: selection of defined deletions in the chromosome of *Corynebacterium glutamicum*. Gene 145: 69-73. https://doi:10.1016/0378-1119(94)90324-7
